# SOHLH2 is essential for synaptonemal complex formation during spermatogenesis in early postnatal mouse testes

**DOI:** 10.1038/srep20980

**Published:** 2016-02-12

**Authors:** Miree Park, Youngeun Lee, Hoon Jang, Ok-Hee Lee, Sung-Won Park, Jae-Hwan Kim, Kwonho Hong, Hyuk Song, Se-Pill Park, Yun-Yong Park, Jung Jae Ko, Youngsok Choi

**Affiliations:** 1 Department of Biomedical Science, CHA University, Seongnam-si, Gyeonggi-do 463-400, Republic of Korea; 2 Department of Nanobiomedical Science & BK21 PLUS NBM Global Research Center for Regenerative Medicine, Dankook University, Cheonan 330-714, Republic of Korea; 3 Department of Animal Biotechnology, Konkuk University, Seoul 143-701, Republic of Korea; 4 Department of Biotechnology, College of Applied Life Science, Jeju National University, Jeju-do 690-756, Republic of Korea; 5 ASAN Institute for Life Sciences, ASAN Medical Center, Department of Convergence Medicine, University of Ulsan College of Medicine, Seoul 138-736, Republic of Korea

## Abstract

Spermatogenesis- and oogenesis-specific helix-loop-helix transcription factor 2 (SOHLH2) is exclusively expressed in germ cells of the gonads. Previous studies show that SOHLH2 is critical for spermatogenesis in mouse. However, the regulatory mechanism of SOHLH2 during early spermatogenesis is poorly understood. In the present study, we analyzed the gene expression profile of the *Sohlh2*-deficient testis and examined the role of SOHLH2 during spermatogenesis. We found 513 genes increased in abundance, while 492 genes decreased in abundance in 14-day-old *Sohlh2*-deficient mouse testes compared to wildtype mice. Gene ontology analysis revealed that *Sohlh2* disruption effects the relative abundance of various meiotic genes during early spermatogenesis, including *Spo11*, *Dmc1, Msh4, Prdm9, Sycp1, Sycp2, Sycp3, Hormad1*, and *Hormad2*. Western blot analysis and immunostaining showed that SYCP3, a component of synaptonemal complex, was significantly less abundant in *Sohlh2*-deficient spermatocytes. We observed a lack of synaptonemal complex formation during meiosis in *Sohlh2*-deficient spermatocytes. Furthermore, we found that SOHLH2 interacted with two E-boxes on the mouse *Sycp1* promoter and *Sycp1* promoter activity increased with ectopically expressed SOHLH2. Taken together, our data suggest that SOHLH2 is critical for the formation of synaptonemal complexes via its regulation of *Sycp1* expression during mouse spermatogonial differentiation.

The spermatogenesis- and oogenesis-specific helix-loop-helix transcription factor 2 (SOHLH2) was first identified as a germ cell-specific factor^1^. SOHLH2 is expressed in pre-meiotic germ cells in the testis and ovary^1^,^2^,^3^. The disruption of *Sohlh2* results in infertility due to a defect in spermatogenesis and oogenesis^2^,^3^,^4^. *Sohlh2*-deficient testes look normal at birth, but they lose germ cells rapidly during postnatal development. *Sohlh2* knockout (KO) mice show fewer spermatogonia and spermatocytes by postnatal day 7. In adult *Sohlh2* KO males, testis weight is on average 3 to 4 times less than that of wild type (WT) mice, and there are no detectable spermatids in the seminiferous tubules^3^,^4^. During early spermatogenesis, *Sohlh2* KO mice have lower numbers of intermediate and type B spermatogonia than undifferentiated type A spermatogonia^3^,^4^. As SOHLH2 regulates the transcription of several genes such as *Kit, Zp*, and *Ngn3* through the DNA binding element E-box^5^,^6^, SOHLH2 may be a critical component of a regulatory network initiating differentiation of spermatogonia into spermatids during the meiotic process. However, little is known about the potential role of SOHLH2 in the meiotic process during spermatogenesis.

In this study, we investigated the effect of SOHLH2 on gene expression by testing for differences in testicular gene expression profiles between WT and *Sohlh2* KO mice. We found that several meiotic factors were significantly decreased in the *Sohlh2*-deficient testes. In addition, we showed that the regulation of meiotic factors by SOHLH2 is crucial for synaptonemal complex formation during spermatogenesis.

## Results

### Defect of early spermatogenesis in *Sohlh2*-deficient testes

**Figure 1:**
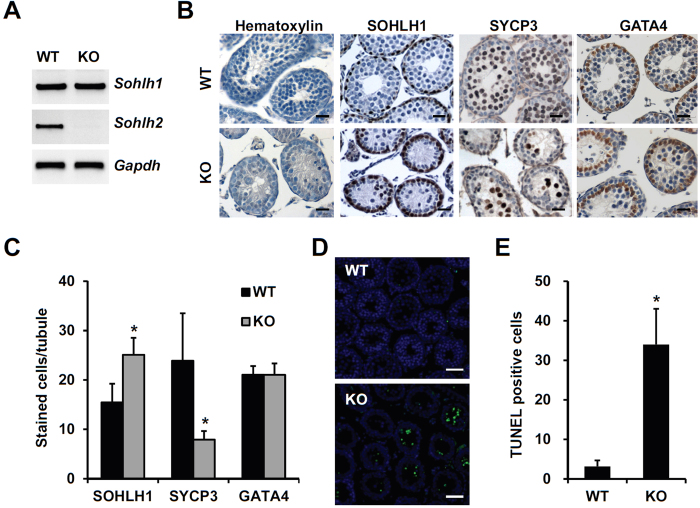
Defect of spermatogenesis in *Sohlh2* KO testes during early postnatal development. (**A**) A representative image of RT-PCR analysis showing the deficiency of *Sohlh2* expression in 2-week-old *Sohlh2* KO mice. Total RNA was isolated from WT and *Sohlh2* KO testes. RT-PCR was performed using primers for *Sohlh1*, *Sohlh2*, and *Gapdh* mRNA. (**B**) Immunohistochemical analysis demonstrating a defect of spermatogenesis in *Sohlh2* KO testes during early postnatal development. Sections of WT and *Sohlh2* KO testes were stained with hematoxylin or immunostained with antibodies. Anti-SOHLH1, anti-SYCP3, and anti-GATA4 antibodies were used to stain spermatogonia, spermatocyte, and sertoli cell, respectively. Testes were prepared from 2-week-old WT and *Sohlh2* KO mice. Scale bars represent 25 μm. (**C**) SOHLH2-, SYCP3-, and GATA4-positive cell numbers in 2-week-old *Sohlh2* KO versus WT mice. The number of positively stained cells per tubule was determined in the cross section of WT and *Sohlh2* KO testes. Each group contained three mice. A minimum of 45 tubules per testis were counted for the cell number analysis. Data are presented as the mean ± SD. *p < 0.01 versus WT. (**D**) TUNEL assay showing the apoptotic cells in testes sections from WT and *Sohlh2* KO mice. Apoptotic cells (green) were frequently detected in *Sohlh2* KO testes. DAPI staining was used to demonstrate cellular nuclei. (**E**) Average number of TUNEL-positive cells in WT and *Sohlh2* KO testes (40 × 40 μm^2^ area). *p < 0.01 versus WT, *n* = 8.

To investigate SOHLH2 effect on early spermatogenesis, we used 2-week-old WT and *Sohlh2* KO mice. The deficiency of *Sohlh2* expression in KO mice was confirmed by RT-PCR analysis (Fig. 1A). Histological analysis showed an abnormal spermatogenesis due to a reduced cell number in the seminiferous tubules of KO testes (Fig. 1B). Cell types in the seminiferous tubules were examined by immunohistochemistry using the cell type-specific markers; SOHLH1 for spermatogonia, SYCP3 for spermatocytes, and GATA4 for sertoli cells. The numbers of SOHLH1-positive cells were increased in KO testes and the numbers of GATA4-positive cells in KO testes were similar to that in WT testes (Fig. 1B,C). By contrast, SYCP3-positive spermatocytes were significantly reduced in KO testes compared with WT testes (Fig. 1B,C). These findings suggest that abnormal spermatogenesis in *Sohlh2*-deficient testes during early postnatal development is caused by disruption of spermatocytes but not by abnormality of spermatogonia or sertoli cells. To further investigate that the potential disruption of spermatocytes is related with cell death, we performed cell death analysis using a terminal deoxynucleotidyl transferase mediated dUTP-biotin nick end labeling (TUNEL) assay. TUNEL-positive cells were significantly increased in KO testes (Fig. 1D,E). These data suggest that differentiation of spermatogonia to early spermatocytes can be occurred in *Sohlh2*-knockout testes during early postnatal development. Therefore, *Sohlh2*-deficiency may lead to the apoptosis of early prophase I spermatocytes, resulting in abnormal spermatogenesis.

### Effect of *Sohlh2*-deficiency on gene expression in the testes

**Figure 2:**
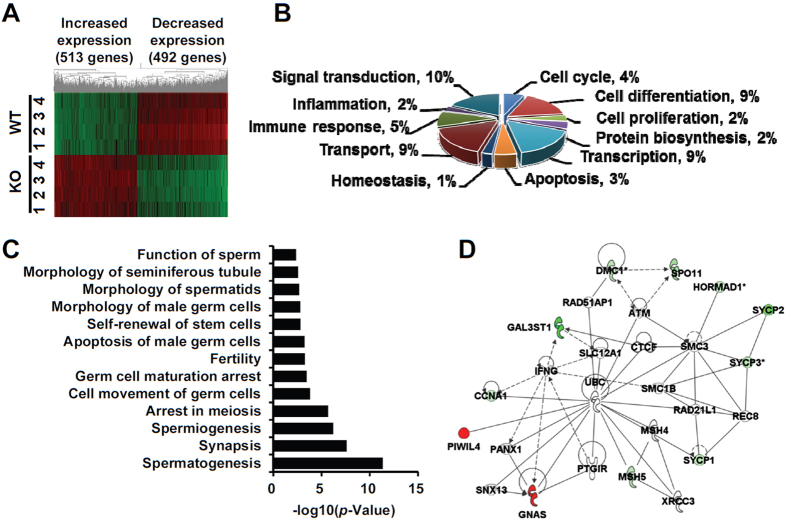
Gene expression profiles in wildtype and *Sohlh2*-deficient mouse testes. (**A**) Heat map of gene profile analysis. Each column represents the relative expression of each gene from different WT (*n* = 4) and *Sohlh2* KO (*n* = 4) testes. There are 513 genes increased and 492 genes decreased by *Sohlh2*-deficiency. (**B**) Gene ontology analysis of 11 different functional categories. The genes were separated into groups using gene ontology and are presented as percentage of total genes altered by *Sohlh2* KO. (**C**) The genes altered by *Sohlh2*-deficiency were categorized based on the processes related with male reproductive system. (**D**) Gene functional association networks for the genes that are involved in meiosis. The intensity of the node color indicates the degree of up-regulation (red) or down-regulation (green).

To characterize which genes are affected by *Sohlh2*-deficiency, we performed microarray analysis using *Sohlh2* KO and WT testes when spermatogonia entered meiosis in 2-week-old mice. We discovered at least a 2-fold increase or decrease in the expression of 1005 genes in the KO testes (Fig. 2A). Among these genes, 513 were increased (Supplementary Table S1) and 492 were decreased in their mRNA expression (Supplementary Table S2). Based on gene ontology analysis (Fig. 2B), we found that many of these genes are involved in cell differentiation (e.g., *Runx3, Kit* and *Piwil1*), signal transduction (e.g., *Dll3, Jag2*, and *Prok2*), transport (e.g., *slc9a6* and *Cebpe*), transcription (e.g., *Tbx1, Ybx2*, and *Lin28b*), apoptosis (e.g., *Aifm3, Scp2*, and *Scp3*), proliferation (e.g., *Kit* and *Ppar-gamma*), protein biosynthesis (e.g., *Nmt2* and *Paip1*), homeostasis, and inflammation. We also observed that *Sohlh2*-deficiency caused abnormal expression of genes associated with spermatogenesis, spermiogensis, function of sperm, and morphology of seminiferous tubules and spermatids (Fig. 2C and Supplementary Table S3). It was most likely that the defects in gene expression of meiosis factors were related to loss of germ cells in *Sohlh2*-deficient mice (Fig. 2D).

### Expression of meiotic factors in *Sohlh2*-deficient testes

**Figure 3:**
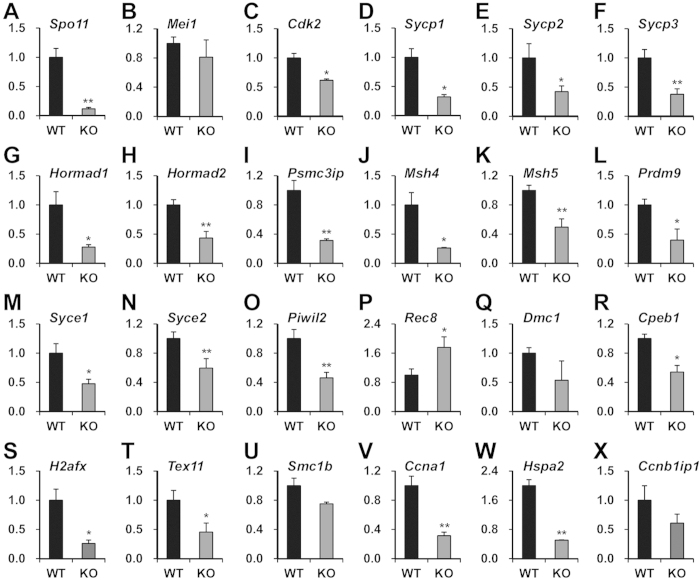
Expression of meiotic factors in *Sohlh2*-deficient testes. Gene expression was quantified by real time-RT-PCR. (**A**,**B**) Expression of leptotene-related genes. (**C**–**Q**) Expression of zygotene- and early pachytene-related genes. (**R–U**) Expression of pachytene-related genes. (**V–X**) Expression of late pachytene- and diplotene-related genes. Data were normalized against *Gapdh* expression and presented as the mean relative quantity ± SEM. *p < 0.05 and **p < 0.01 versus WT.

To confirm *Sohlh2* deficiency-induced changes in the relative abundance of meiotic genes, we carried out quantitative real-time reverse transcription polymerase chain reaction (RT-PCR) using total RNA from *Sohlh2* KO and WT testes of 2-week-old mice. Genes specific to prophase I (i.e., leptotene, zygotene, pachytene and diplotene stages) were selected according to previous studies^7^, and their primers were designed. Of the leptotene-related factors, *Spo11* expression was significantly reduced in KO testes (Fig. 3A), whereas *Mei1* expression was unchanged (Fig. 3B). Of the zygotene- and pachytene-related factors, the expression of *Cdk2*, *Sycp1*, *Sycp2*, *Sycp3, Hormad1*, and *Hormad2* was significantly decreased in KO testes (Fig. 3C,H). Among these genes, *Sycp1*, *Sycp2*, *Sycp3, Hormad1, and Hormad2* are critical for the formation of synaptonemal complexes during meiosis. Other zygotene- and pachytene-related factors, including *Psmc3ip*, *Msh4*, *Msh5*, *Prdm9*, *Syce1*, *Syce2*, and *Piwil2*, were also significantly reduced in the KO testes (Fig. 3I,O). However, *Rec8* expression was significantly increased (Fig. 3P) and *Dmc1* expression was unchanged (Fig. 3Q) in KO testes. The pachytene-related factors *Cpeb1, H2afx*, and *Tex11* were significantly diminished in KO testes (Fig. 3R,T), whereas *Smc1b* expression was unchanged (Fig. 3U). Expression of diplotene-related factors *Ccna1* (Fig. 3V) and *Hspa2* (Fig. 3W) was significantly decreased in KO testes, whereas *Ccnb1ip1* expression was unaffected (Fig. 3X). The gene expression profile and early spermatogenic arrest in *Sohlh2*-deficient mice raise questions on if SOHLH2 is key regulator of meiotic gene expression, which might be crucial for successful meiotic division of germ cells during spermatogenesis.

### Relationship between SOHLH2 and synaptonemal complex protein SYCP1

**Figure 4:**
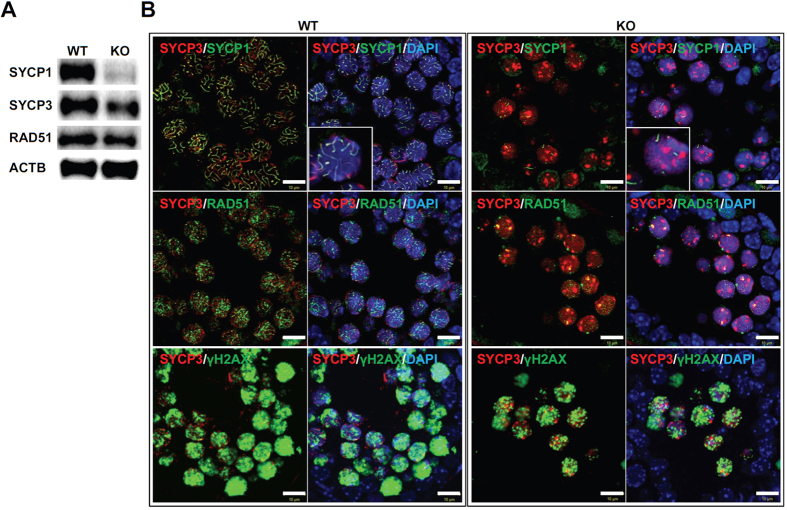
Reduction in SYCP1 protein expression in the *Sohlh2*-deficient testes. (**A**) Western blot analysis for SYCP1, SYCP3, and RAD51 expression in testes. Protein extracts from testes of 2-week-old WT and *Sohlh2* KO mice were used. *β*-actin was used as a loading control. (**B**) Immunostaining showing the localization and distribution of SYCP1, SYCP3, RAD51, and γH2AX in the testes of 2-week-old WT and *Sohlh2* KO mice. The nucleus was stained with DAPI. Scale bars indicate 10 μm. Inset shows higher magnification view.

To examine the protein levels of major meiotic proteins including SYCP1, SYCP3, and RAD51 in the *Sohlh2* KO testis, we performed western blot and immunofluorescence analyses. The expression of SYCP1, which is a transverse element in the synaptonemal complex, was significantly reduced in KO testes (Fig. 4A). Also, the expression of SYCP3, which is a lateral element in the synaptonemal complex, was reduced in KO testes (Fig. 4A). Consistent with these results, immunofluorescence staining showed a decrease in SYCP1 signal intensity in spermatocytes from KO testes compared with those from WT testes. The signal intensity of SYCP3 was not affected by *Sohlh2* deficiency (Fig. 4B). Instead, the number of SYCP3-positive cells was significantly decreased, affecting SYCP3 expression level in western blot (Fig. 4A). γH2AX is a phosphorylated form of H2AX, which is associated with meiotic DNA double-strand break. DNA double-strand break normally occurs in the nuclei of primary spermatocytes, but not in sertoli cells, spermatogonia, and spermatid. γH2AX is known to be highly detectable in premeiotic S-phase and leptotene stage of spermatocytes^8^. Shown in Fig. 4B, there were no defects in γH2AX staining in *Sohlh2* KO spermatocytes. This suggests that the reduction of synaptonemal complex component, SYCP1, might affect male meiosis through synaptonemal complex formation.

**Figure 5:**
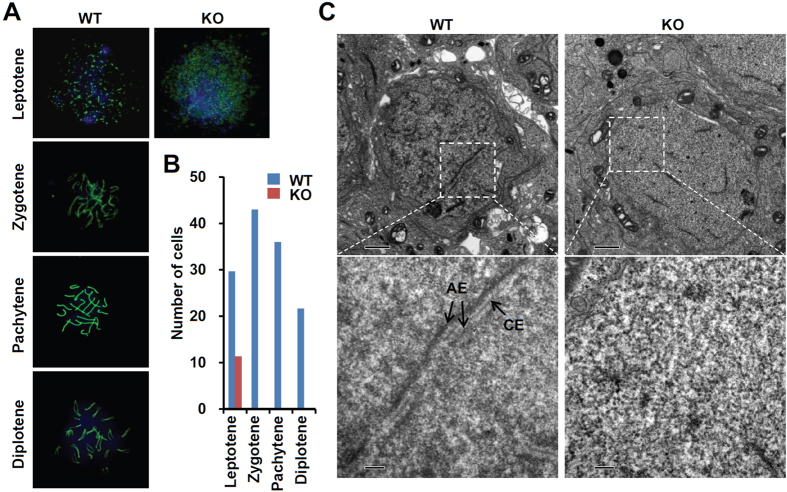
Lack of synapsis of homologous chromosomes in the *Sohlh2*-deficient testes. (**A**) The process of meiosis was analyzed using a chromosome spreading assay. Each substage of prophase I was observed by staining WT and *Sohlh2* KO testes with anti-SYCP3 antibody. DAPI was used to stain the nucleus. (**B**) Numbers of cells at each stage were counted from sections of WT and *Sohlh2* KO testes (*n* = 4 per group). (**C**) The formation of synapses was investigated using transmission electron microscopy of testes from WT and *Sohlh2* KO mice. AE, axial complex; CE, central complex. Inset shows high-power image (75000 × magnification).

Previous studies indicate that *Sycp1* and *Sycp3* deficiency affects chromosome synapsis during meiosis I^9^,^10^. Therefore, we examined whether SOHLH2 that was critical for *Sycp1* expression affects formation of synaptonemal complexes in *Sohlh2*-deficient testes by using SYCP3 staining to examine chromosome structure. In the testes of 2-week-old WT mice, we found normal progression of spermatocytes across leptotene, zygotene, pachytene, and diplotene stages (Fig. 5A), whereas KO spermatocytes were found only in the leptotene stage (Fig. 5A,B). To verify the defect of *Sohlh2*-deficient spermatocytes in synaptonemal complex formation, we investigated the structure of synaptonemal complexes using electron microscopy. Tripartite synaptonemal complex structures were well visualized during meiosis I in WT testes, but not in KO testes (Fig. 5C). These findings indicate that SOHLH2 controls synaptonemal complex formation via regulating *Sycp1* expression.

**Figure 6:**
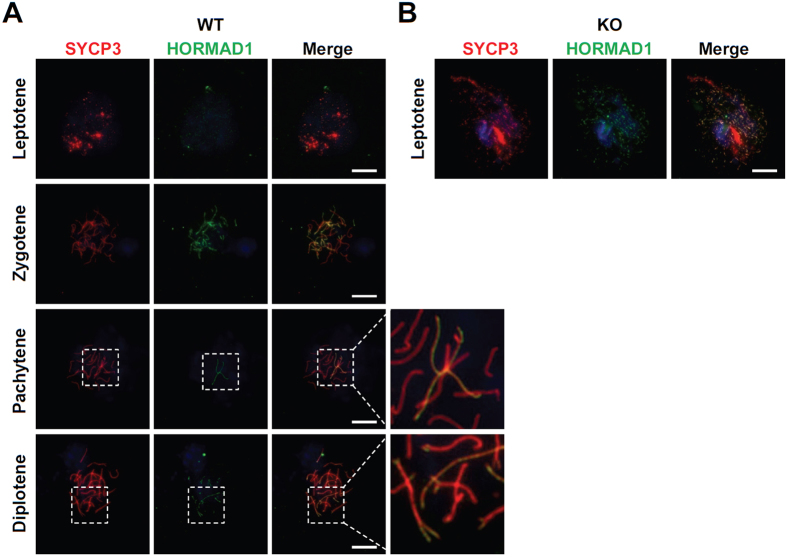
Presence of SYCP3 and HORMAD1 in the *Sohlh2*-deficient testis. (**A**) Localization of HORMAD1 and SYCP3 in WT spermatocytes. DAPI was used for staining of nucleus. Inset shows higher magnification image. At the pachytene stage, HORMAD1 was only detected on unsynapsed chromosomes (i.e., sex chromosomes). (**B**) Localization of HORMAD1 and SYCP3 in *Sohlh2* KO spermatocytes. Scale bars indicate 10 μm.

**Figure 7:**
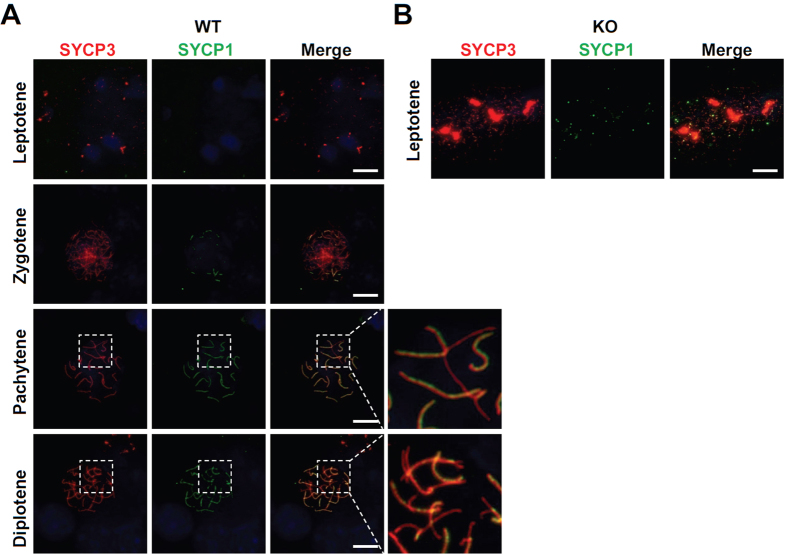
Absence of SYCP1 in the *Sohlh2*-deficient testis. (**A**) Localization of SYCP1 and SYCP3 in WT spermatocytes. Inset shows higher magnification image. DAPI was used to stain nucleus. At pachytene satage, SYCP1 co-localized with SYCP3 except for unsynapsed chromosomes (i.e., sex chromosomes). (**B**) SYCP1 was not present in KO spermatocytes. Scale bars indicate 10 μm.

Next, we examined the localization of HORMAD1, which localizes to unsynapsed chromosome axes during prophase I^11^,^12^. HORMAD1 was gradually detected in unsynapsed chromosome during prophase I in WT spermatocytes (Fig. 6A). HORMAD1 was detected on the sex body complex in pachytene (Fig. 6A), consistent with previous reports^11^,^12^. HORMAD1 signal was detected as dispersed spots in KO spermatocytes (Fig. 6B). Next, we investigated the localization of SYCP1, which is indicative of synapsed chromosomes. In WT testes, SYCP1 localized to synapsed chromosomes only after zygotene stage, at which the synaptonemal complex begin to form (Fig. 7A). At the pachytene stage, SYCP1 signal was detected on synapsed autosomal chromosomes except for the sex chromosome, which showed partial detection due to unsynapsed region on the chromosome (Fig. 7A). However, KO spermatocytes showed dispersed spots of SYCP1 signal (Fig. 7B), similar to the HORMAD1 signal (Fig. 6B). These results suggest that SOHLH2 is very important for the formation of synaptonemal complexes through either direct or indirect regulation of meiotic factors including SYCP1.

### Regulation of *Sycp1* expression by SOHLH2

**Figure 8:**
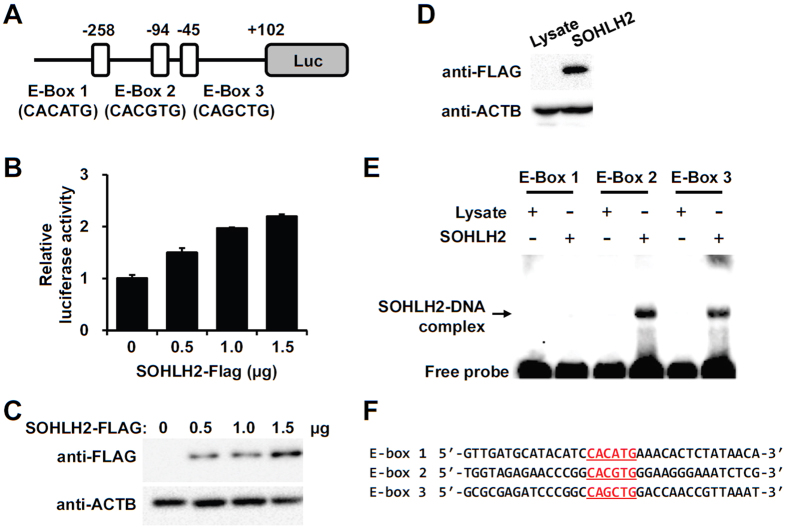
SOHLH2 increases *Sycp1* promoter activity. (**A**) Reporter vector containing the luciferase gene fused with the mouse *Sycp1* promoter region (~572 bp), which possesses three putative E-boxes at −258 (CACATG), −94 (CACGTG), and −45 (CAGCTG) positions. (**B**) Relative luciferase activity was measured by a luminometer in the absence or presence of FLAG-tagged SOHLH2 expression vector. (**C**) Western blot analysis showing SOHLH2 expression in whole cell lysates used in the luciferase assay. FLAG-tagged SOHLH2 was detected using anti-FLAG antibody. β-actin was used as internal control. (**D**) *In vitro*-translated FLAG-tagged SOHLH2 shown by western blot analysis. (**E**) SOHLH2 binding to E-boxes in *Sycp1* promoter. EMSA was performed with three biotin-labeled E-box probes in the absence or presence of *in vitro*-translated SOHLH2. Equal volume of *in vitro* translation reaction mixture containing empty vector was used as control (Lysate). (**F**) The DNA sequences of three E-box probes used in EMSA.

To investigate whether *Sycp1* expression is directly regulated by SOHLH2, we examined *Sycp1* promoter (up to ~572 kb) using a computational program (TFSEARCH). We found three putative bHLH domain-binding elements, E-boxe elements (CANNTG), at −258, −94 and −45 upstream from start codon on the mouse *Sycp1* promoter (Fig. 8A). To examine the effect of SOHLH2 on transactivation of the *Sycp1* promoter, HEK293T cells were transiently transfected with the firefly luciferase reporter vector fused with its promoter containing the three putative E-boxes. Compared with a mock vector, expression of FLAG-tagged SOHLH2 vector resulted in increasing amounts of *Sycp1* promoter activity (Fig. 8B,C). Electrophoretic mobility shift assay (EMSA) showed that SOHLH2 directly bound to E-box sequences containing CACGTG or CAGCTG (Fig. 8D,F). These results suggest that *Sycp1* expression might be directly regulated via two of three putative E-boxes under SOHLH2 regulation during spermatogenesis in mouse.

## Discussion

The process of spermatogenesis in the seminiferous tubules is a complex biological event. Spermatogonial stem cells perpetually proliferate and differentiate into sperm cells through meiosis, and this process is highly regulated by germ-cell-specific factors and hormones^13^. In a previous study, we discovered that SOHLH2 is a germ cell transcription factor expressed in the gonad^1^. SOHLH2 plays a role in the differentiation of mature spermatogonia during spermatogenesis^3^,^4^ as well as in oogenesis^2^. *Sohlh2* deficiency disrupts normal spermatogenesis by altering the expression of many genes in the testis. However, the mechanism by which SOHLH2 regulates gene expression was unclear. Recently, two research groups showed that SOHLH2 directly regulates several gene targets including *Kit*, *Gfra1*, and *Sox3*^5^,^6^. In particular, SOHLH2 binds independently to the E-box on the *Kit* promoter and stimulates *Kit* expression with SOHLH1, another germ cell-specific transcriptional factor^14^. Both SOHLH2 and SOHLH1 play crucial roles in the differentiation of spermatogonial stem cells^3^,^4^,^14^. However, these findings are not sufficient to fully explain molecular events mediated by SOHLH2 that are essential for spermatocytes to develop normally from differentiating spermatogonia.

Stem and early progenitor spermatogonia give rise to differentiating spermatogonia that complete meiosis to produce haploid spermatogenic cells termed spermatids. SOHLH1 and SOHLH2 are co-expressed in undifferentiated and differentiating spermatogonia, but not in primitive undifferentiated spermatogonia expressing glial cell-derived neurotrophic factor receptor alpha I^6^. In previous study, accumulation of STRA8-positive differentiated spermatogonia was previously shown in *Sohlh2*-deficient testes^5^,^6^, suggesting that differentiation of spermatogonia does not occur properly. SOHLH2 binds directly to chromatin upstream of genes that are essential for spermatogonial stem cell maintenance and spermatogonial differentiation, such as *Gfra1*, *Sox3*, *Sohlh1*, *Sohlh2*, and *Kit*^6^. In our immunohistochemistry, we also found that SOHLH1-positive spermatogonia were more in 2-week-old *Sohlh2* KO testes than in WT testes, suggesting that *Sohlh2*-deficiency caused an impairment in spermatogonial differentiation and resulted in accumulation of SOHLH1-positive spermatogonia. Although *Sohlh2*-deficiency compromised the differentiation of spermatogonia, SYCP3-positive spermatocytes were detectable in KO testes. We propose that early postnatally, *Sohlh2*-deficient spermatogonia are entering meiosis to form abnormally developed leptotene spermatocyte-like germ cells. These data are similar to the previous data showing *Sohlh2* KO testes contain a portion of cells with thread-like chromosome condensation which is a characteristic of leptotene spermatocytes^6^, although premature HORMAD1-positive meiotic cells abnormally appears in 1-week-old *Sohlh2* KO testes. Therefore, we focused on the time when meiosis begins, which is around postnatal day 10 to 14 in mice, to determine whether SOHLH2 regulates the meiotic process. When we examined the gene profiles of testes from 2-week-old male *Sohlh2* KO mice, we found that numerous genes were regulated at this stage, including 1005 genes with at least 2-fold changes in expression relative to WT mice. Many of these genes were involved in meiosis, including *Spo11, Sycp1, Sypc3*, and *Hormad1*. This altered expression of meiotic genes can be derived from the direct effect of *Sohlh2*-deficiency on transcriptional rate of the genes. In addition, a loss of meiotic cells in *Sohlh2* KO testes can result in the reduced mRNA levels due to the vast majority of leptotene to diplotene spermatocyte populations not being present in *Sohlh2*-deficient mice. According to our RT-PCR and immunohistochemistry analysis, it is likely that the decreased level of *Sycp3*mRNA originated from spermatocytes absent after leptonema, rather than *Sohlh2* deficiency-induced down-regulation of gene transcription. In 1-week-old testes, *Spo11* expression was not affected by *Sohlh2*-deficiency^6^, but its expression was drastically decreased in 2-week-old KO testes. *Spo11* is normally expressed at very low level in 1-week-old testes and increased from postnatal day 12. *Spo11* mRNA is detected at the highest level in pachytene spermatocytes^15^. Therefore, the decreased *Spo11* expression might also be a consequence of SOHLH2-induced loss of pachytene cells in KO testes, although more extensive analysis is needed.

**Figure 9:**
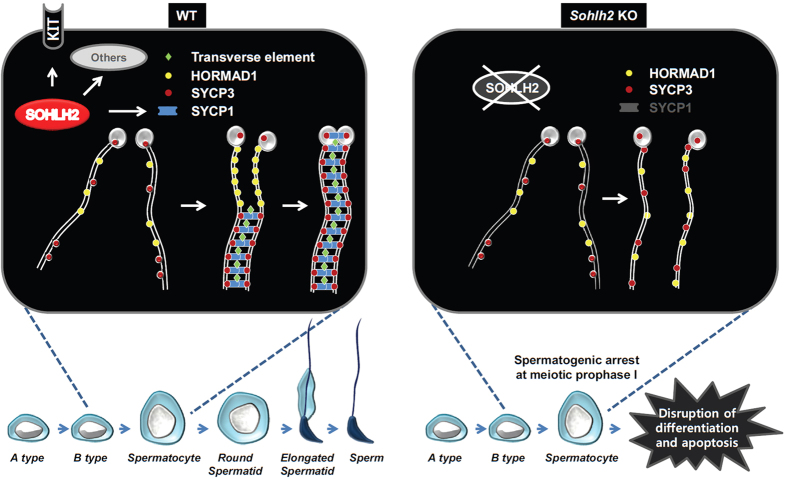
Working model for a role of SOHLH2 in spermatogenesis via regulation of *Sycp1* expression. SOHLH2 acts as a master regulator of target genes including *Kit* during spermatogonial differentiation. In the testes, SOHLH2 is hypothesized to regulate gene expressions critical for meiosis during spermatogenesis. SOHLH2 deficiency leads to spermatogenic arrest early during meiotic prophase I due to defects in components of the synaptonemal complex such as SYCP1, leading to the disruption of differentiation and the apoptosis of spermatocytes.

During meiosis, crossover between homologous chromosomes occurs during prophase I, which consists of substages: leptotene, zygotene, pachytene, and diplotene. Chromosomes are marked for double-strand break during leptotene stage, and they begin to synapse at the zygotene stage. The synapse is completed at the pachytene stage, and the synapsed chromosomes separate with one or two synapsed regions at the diplotene stage. The successful synapse of homologous chromosomes depends on the formation of the synaptonemal complex, for which SYCP1, SYCP2, SYPC3, and HORMAD1 are critical factors. SYCP1 acts as a transverse element in the synaptonemal complex^9^ that bridges between homologous chromosomes, and SYCP2 and SYCP3 are lateral elements of the complex that are localized on the sister chromatids (Fig. 9). HORMAD1 is involved in the double-strand break and synaptonemal complex formation during meiosis and it localizes to unsynapsed chromosome axes during prophase I^11^,^12^,^16^. We found that SYCP1 expression was significantly decreased in *Sohlh2* KO testes. The expression level of SYCP3 in KO spermatocytes was similar to that in WT cells, although the number of SYCP3-positive cells were reduced in KO testes. A chromosome spreading assay showed that *Sohlh2* KO spermatocytes were arrested in leptotene stages, and electron microscopy showed that synaptonemal complexes failed to form in KO spermatocytes. In the study of *Sycp1* function, spermatogenic differentiation is interrupted predominantly at pachytene stage of spermatocytes in *Sycp1* KO testes, suggesting *Sycp1*-deficient spermatocyte can undergo meiosis from leptotene to pachytene^9^. However, *Sohlh2* deficiency is more severe defect in meiosis progression than *Sycp1* deficiency. These results indicate that SOHLH2 plays a role in regulation of SYCP1 expression and the lack of SYCP1 with more additional factors in *Sohlh2* KO testes was involved in misregulated spermatogenesis.

SYCP1 is a major component of transverse elements in the synaptonemal complex. Spermatocytes in *Sycp1* KO testes are arrested during early meiosis^9^, similar to what is observed in *Sohlh2* KO mice. In spite of the importance of SYCP1 in the formation of the synaptonemal complex, little is known about how SYCP1 is regulated during the meiotic process. A previous study investigating the regulation of the *Sycp1* promoter region (up to 2 kb) in gonad showed that a short fragment encompassing the transcription site (−54 to +102) was critical for temporal and spatial regulation during meiosis in male mice^17^. As the region contains an E-box (CAGCTG) at position −45 relative to the transcription start site^17^, this suggests that *Sycp1* expression is regulated by a transcription factor containing a bHLH by way of an E-box on its promoter. When the activity of longer promoter fragment (−260 to +102) was compared with the activity of the short fragment (−54 to +102), expression from the longer promoter fragment was much higher in testes^17^, suggesting the possibility that other promoter regions of *Sycp1* were involved in regulation of its promoter activity. In the present study, we found two additional E-boxes on the *Sycp1* promoter region (−258 and −94) (Fig. 8). SOHLH2 directly bound to two E-boxes at positions −94 and −45 on *Sycp1* promoter and induced *Sycp1* promoter activity. Although a mutation study for SOHLH2-induced *Sycp1* promoter activity remains, we propose that SOHLH2 might regulate the expression of *Sycp1* via binding to two of E-boxes (CACGTG and CAGCTG).

In conclusion, our gene expression profiling identified putative regulatory targets of SOHLH2 as a master transcription factor during spermatogenesis. Furthermore, we revealed that SOHLH2 is associated with progression of meiosis by regulating *Sycp1* expression during spermatogenesis.

## Methods

### Animals

All mouse experiments were carried out in a 129S7/SvEvBrd × C57BL/6 mixed background. *Sohlh2* heterozygous mice were bred to generate *Sohlh2* KO mice as previously described^2^. The care of mice and experimental procedures complied with the Guide for the Care and Use of Laboratory Animals and were approved by the Institutional Agricultural Animal Care and Use Committee of CHA University.

### Histology and immunostaining

Testes were fixed in 4% paraformaldehyde (PFA) and embedded in paraffin wax for histological analysis. After embedding in paraffin, sections (5-μm thick) were cut and mounted on Superfrost Plus slides (Fisher Scientific, USA). For histology, sections were stained with Harris hematoxylin (Sigma-Aldrich, USA) for 5 minutes (min) and washed in running tap water for 5 min.

For immunofluorescence assay, deparaffinized slides were placed in a container with antigen retrieval buffer (10mM sodium citrate, 0.05% Tween 20, pH6.0). The containers were heated in a microwave at full power for 5 min. After boiling, the slides were reheated at 50% power for 5 min. The slides were then cooled to room temperature for 15 min and washed three times for 5 min in distilled water. After permeabilization in a buffer solution (0.2% Triton X−100 in phosphate-buffered saline (PBS)) at room temperature for 45 min, the slides were washed with PBS and incubated with blocking buffer (PBS with 10% bovine serum albumin (BSA)) in a humidified chamber for 1 hour at room temperature. The blocked slides were incubated with primary antiboides at 4 °C for overnight. The primary antibodies were anti-GATA4 (1:500; Abcam), anti-HORMAD1 (1:500)^17^, anti-SYCP1 (1:750; Abcam), anti-SYCP3 (1:750; Santa Cruz), anti-RAD51, and anti-γH2AX (1:500; Cell Signaling). After three times washes with PBS for 5 minutes, the sections were incubated with the secondary antibody in blocking buffer for 2 hours at room temperature. The secondary antibodies (Invitrogen, USA) conjugated with Alexa Fluor^®^ 488 or Alexa Fluor^®^ 594 were used. The slides were washed three times with PBS, and DAPI (4′,6-Diamidino-2-Phenylindole, 1:20000; Life Technologies, USA) was added. Finally, mounting Medium (Dako, USA) was applied, and a glass coverslip was placed over the sections.

For immunohistochemical staining, deparaffinized slides were incubated in 0.3% H_2_O_2_ in PBS and 100% methanol for 15 min to quench endogenous peroxidase activity after antigen retrieval. The slides were incubated with a blocking buffer (10% normal serum with 10% BSA) for 2 hours at room temperature. After tapping off the blocking agent, biotin/avidin blocking solutions (Vector Labs, USA) were added to block endogenous biotin and avidin for 15 min. Primary antibodies were applied as described above. On the next day, the sections were further processed with an avidin-biotin-horseradish peroxidase complex (Vector Labs, USA). The peroxidase signals were developed with a 0.01% 3,3′-diaminobenzidine solution (Vector Labs) according to the manufacturer’s protocol and counterstained with hematoxylin. The slides were rehydrated with ethanol and xylene, and mounted with Permount mounting medium (Fisher Scientific, USA).

### TUNEL assay

Analysis of TUNEL staining was performed using a TUNEL assay kit (Roche, USA) according to the manufacturer’s instructions. TUNEL-positive cells in testis were counted under microscopy.

### Analysis of gene expression profile

The testes were separated from 2-week-old WT and KO mice (n = 4 per group), and total RNA was extracted using an RNeasy mini kit (Qiagen, Germany). A 12-bay MAUI hybridization system was used for the Agilent mouse whole genome 8 × 60 K array platform (Agilent, USA). Image and signal data extraction was performed using Agilent Scanner, Feature Extract v10.7.3.1. Because four separate experiments were performed on WT and KO RNA, signal intensities for particular genes were averaged across experiments, and the ratio of WT- to-KO signal was calculated. Data were normalized using the robust multi-array average method^18^. Differentially expressed gene selection, clustering, and functional analysis were performed by GenoCheck (Korea).

### Real-time RT-PCR

Total RNAs were converted to cDNA using iScript cDNA Synthesis kit (Bio-Rad Laboratories, USA). Quantitative real-time PCR was performed using QuantiTect SYBR Green PCR reagents (Qiagen, Germany) and iCycler (Bio-Rad, USA). The primers used for the PCR were listed in supplementary Table S4. Results were evaluated with iQ5^TM^Optical system software. RNA expression level was normalized to *Gapdh* mRNA level and calculated using the relative quantification approach based on the ΔΔCt method . The relative amount of cDNA was determined as 2^−ΔΔCt^.

### Chromosome spreading assay

Testes were removed from 2-week-old mice and incubated in trypsin-EDTA solution with DNas1 at 37 °C for 10 min and washed in PBS. Trypsinized testes were pipetted repeatedly and filtered by mesh (70 μm) to remove debris. After centrifugation, cells were resuspended in 1 ml PBS. Suspended cells were placed on poly-L-lysine-coated slides containing sucrose solution (40 mg/ml) and treated with permeabilization solution containing 0.005% Triton X-100. The slides were fixed in 2% PFA and 0.02% SDS for 1 hour at room temperature. The incubated slides were washed six times in distilled water, dried briefly, and stored at −80 °C until use.

### Western blot analysis

Protein samples were loaded on SDS-PAGE (4–20% gradient gel; Invitrogen), transferred to nitrocellulose membranes and blocked with 5% non-fat milk in tris-buffered saline (TBS) containing 0.1% Tween 20 (TBS-T) for 1 hour at room temperature. The membrane was incubated with specific primary antibody diluted in TBS-T/5% milk at 4 °C overnight followed by appropriate HRP-conjugated secondary antibody (Invitrogen) diluted at 1:1000 in TBS-T/5% milk for 1 hour at room temperature. The membrane was developed using the ECL Western Blotting substrate kit (Gendepot, USA). The relative expression of protein was analyzed using the ChemiDoc XRS system (Bio-Rad, USA).

### Electron microscopy

Testes were fixed with 2% formaldehyde and 3% glutaraldehyde, for 2 hour at room temperature. Samples were treated with 0.5% uranyl acetate and osmium tetraoxide, dehydrated with ethanol, and embedded in LX−112 medium. The tissue was polymerized in a 70 °C oven for 2 days and then cut into ultrathin sections (70–100 nm) with an ultracut microtome. The sections were stained in 1% aqueous uranyl acetate and placed for 2 min in 1% aqueous lead citrate at room temperature in an M Stainer, The sections were then examined using a transmission electron microscope.

### Luciferase assay

To measure SOHLH2-induced *Sycp1* promoter activity, a 572-bp fragment of *Sycp1* promoter ([−470/+ 102] promoter) encompassing three putative E-boxes and transcription start site was PCR-amplified using the primers 5′-CTCGAGAGCACAGGACTCATGTTTGG-3′ and 5′-AAGCTTCGTGACAGAGGTGTGTACGTG-3′. The fragment was first cloned into pTOP TA V2 (Enynomics, Korea) and a 572 bp *Hind*III-*Xho*I fragment was subcloned into pGL4.10-luciferase vector (Promega, USA). The DNA fragment encoding SOHLH2 was synthesized using cDNA from mouse testes and cloned to pCMV-Tag 2A vector (Agilent Technologies, USA) to make FLAG-tagged SOHLH2 expression vector. HEK293T cells were seeded into 6-well plates at a density of 6 × 10^5^ cells/well 24 hour prior to transfection. Cells were co-transfected with the indicated amount of SOHLH2 expression vector, 200 ng of *Sycp1* promoter luciferase plasmid, and 50 ng of pRL-TK plsmid (Promega) using Lipofectamine 2000 (Invitrogen). After 2 days, cells were harvested, lysed, and analyzed for luciferase activity using dual-luciferase reporter assay system (Promega) and Centro XS^3^ LB960 microplate luminometer (Berthod Technologies, USA).

### Electrophoretic mobility shift assay

For *in vitro* translation of SOHLH2, DNA encoding FLAG-tagged SOHLH2 was subcloned into pcDNA3.1/Hygro vector (Invitrogen). FLAG-tagged SOHLH2 was *in vitro* translated using TNT^®^ quick coupled transcription/translation system (Promega) according to manufacturer’s instructions. Successful translation of SOHLH2 was confirmed by western blot analysis. One microliter of protein products was electrophoresed through 10% SDS-polyacrylamide gel and transferred into nitrocellulose membrane. FLAG-tagged SOHLH2 was visualized by incubation of anti-FLAG antibody conjugated with HRP (Sigma-Aldrich) and the ECL kit. Biotin 3′ end-labeled complementary oligonucleotides containing putative E-boxes were obtained from Macrogen (Korea) and annealed by heating at 95 °C for 10 min and slowly cooling to room temperature in a buffer of Tris (10mM, pH 8), EDTA (1 mM, pH 8), and NaCl (100 mM). To form the double-stranded biotin probe-protein complex, we used LightShift EMSA optimization kit (Thermo Scientific, USA). Briefly, 2 μl of *in vitro* translated protein products and 50 fmol of biotin probes were incubated in 1 × binding buffer (Thermo Scientific) containing 2.5% glycerol, 100 mM MgCl_2_, 50 ng/μl poly(dI·dC), and 0.05% NP-40 at room temperature for 20 min. The mixture was separated at 10 volts/cm through 0.5 × TBE−6% polyacrylamide gel and then transferred into Hybon-XL membrane using semi-dry transfer cell (Bio-Rad, USA) at 0.4 A for 1 hour. The DNA was cross-linked to the membrane using Spectrolinker XL−1000 UV crosslinker (Spectronics corporation, USA). Biotin-labeled probes were detected using Chemiluminescent nucleic acid detection module (Thermo Scientific) according to the manufacturer’s instructions. Image was acquired using the ChemiDoc XRS system (Bio-Rad).

### Statistics

The data from real time RT-PCR, immunohistochemistry, and TUNEL assay were analyzed using a two-tailed Student’s *t* test. Data are expressed as the mean ± standard deviation (SD) or mean ± standard error of the mean (SEM).

## Additional Information

**How to cite this article**: Park, M. *et al*. SOHLH2 is essential for synaptonemal complex formation during spermatogenesis in early postnatal mouse testes. *Sci. Rep*. **6**, 20980; doi: 10.1038/srep20980 (2016).

## Supplementary Material

Supplementary Table S1

Supplementary Table S2

Supplementary Table S3

Supplementary Table S4

## References

[b1] Ballow D. J., Xin Y., Choi Y., Pangas S. A. & Rajkovic A. Sohlh2 is a germ cell-specific bHLH transcription factor. Gene Expr Patterns 6, 1014–1018 (2006). 16765102 10.1016/j.modgep.2006.04.007

[b2] Choi Y., Yuan D. & Rajkovic A. Germ cell-specific transcriptional regulator sohlh2 is essential for early mouse folliculogenesis and oocyte-specific gene expression. Biol Reprod 79, 1176–1182 (2008). 18753606 10.1095/biolreprod.108.071217 PMC2780471

[b3] Toyoda S. . Sohlh2 affects differentiation of KIT positive oocytes and spermatogonia. Dev Biol 325, 238–248 (2009). 19014927 10.1016/j.ydbio.2008.10.019

[b4] Hao J. . Sohlh2 knockout mice are male-sterile because of degeneration of differentiating type A spermatogonia. Stem Cells 26, 1587–1597 (2008). 18339773 10.1634/stemcells.2007-0502

[b5] Barrios F. . SOHLH1 and SOHLH2 control Kit expression during postnatal male germ cell development. J Cell Sci 125, 1455–1464 (2012). 22328502 10.1242/jcs.092593

[b6] Suzuki H. . SOHLH1 and SOHLH2 coordinate spermatogonial differentiation. Dev Biol 361, 301–312 (2012). 22056784 10.1016/j.ydbio.2011.10.027 PMC3249242

[b7] Handel M. A. & Schimenti J. C. Genetics of mammalian meiosis: regulation, dynamics and impact on fertility. Nat Rev Genet 11, 124–136 (2010). 20051984 10.1038/nrg2723

[b8] Mahadevaiah S. K. . Recombinational DNA double-strand breaks in mice precede synapsis. Nat Genet 27, 271–276 (2001). 11242108 10.1038/85830

[b9] de Vries F. A. . Mouse Sycp1 functions in synaptonemal complex assembly, meiotic recombination, and XY body formation. Genes Dev 19, 1376–1389 (2005). 15937223 10.1101/gad.329705 PMC1142560

[b10] Yuan L. . The murine SCP3 gene is required for synaptonemal complex assembly, chromosome synapsis, and male fertility. Mol Cell 5, 73–83 (2000). 10678170 10.1016/s1097-2765(00)80404-9

[b11] Fukuda T., Daniel K., Wojtasz L., Toth A. & Hoog C. A novel mammalian HORMA domain-containing protein, HORMAD1, preferentially associates with unsynapsed meiotic chromosomes. Exp Cell Res 316, 158–171 (2010). 19686734 10.1016/j.yexcr.2009.08.007

[b12] Wojtasz L. . Mouse HORMAD1 and HORMAD2, two conserved meiotic chromosomal proteins, are depleted from synapsed chromosome axes with the help of TRIP13 AAA-ATPase. PLoS Genet 5, e1000702 (2009). 19851446 10.1371/journal.pgen.1000702 PMC2758600

[b13] Matzuk M. M. & Lamb D. J. The biology of infertility: research advances and clinical challenges. Nat Med 14, 1197–1213 (2008). 18989307 10.1038/nm.f.1895 PMC3786590

[b14] Ballow D., Meistrich M. L., Matzuk M. & Rajkovic A. Sohlh1 is essential for spermatogonial differentiation. Dev Biol 294, 161–167 (2006). 16564520 10.1016/j.ydbio.2006.02.027

[b15] Shannon M., Richardson L., Christian A., Handel M. A. & Thelen M. P. Differential gene expression of mammalian SPO11/TOP6A homologs during meiosis. FEBS letters 462, 329–334 (1999). 10622720 10.1016/s0014-5793(99)01546-x

[b16] Shin Y. H. . Hormad1 mutation disrupts synaptonemal complex formation, recombination, and chromosome segregation in mammalian meiosis. PLoS Genet 6, e1001190 (2010). 21079677 10.1371/journal.pgen.1001190 PMC2973818

[b17] Sage J. . Temporal and spatial control of the Sycp1 gene transcription in the mouse meiosis: regulatory elements active in the male are not sufficient for expression in the female gonad. Mech Dev 80, 29–39 (1999). 10096061 10.1016/s0925-4773(98)00191-9

[b18] Irizarry R. A. . Exploration, normalization, and summaries of high density oligonucleotide array probe level data. Biostatistics 4, 249–264 (2003). 12925520 10.1093/biostatistics/4.2.249

